# Community-based knowledge transfer and exchange: Helping community-based organizations link research to action

**DOI:** 10.1186/1748-5908-5-33

**Published:** 2010-04-27

**Authors:** Michael G Wilson, John N Lavis, Robb Travers, Sean B Rourke

**Affiliations:** 1 Health Research Methodology Program, Department of Clinical Epidemiology and Biostatistics, McMaster University 1200 Main Street West, Hamilton, ON, Canada; 2 Ontario HIV Treatment Network, 1300 Yonge St,, Suite 600, Toronto, ON, Canada; 3 McMaster Health Forum, McMaster University, 1280 Main Street West, L417, Hamilton, ON, Canada; 4 Centre for Health Economics and Policy Analysis, McMaster University, 1280 Main Street West, Hamilton, ON, Canada; 5 Department of Clinical Epidemiology and Biostatistics, McMaster University 1200 Main Street West, Hamilton, ON, Canada; 6 Department of Political Science, McMaster University, 1280 Main St. West, Hamilton, ON, Canada; 7 Department of Psychology, Wilfrid Laurier University, Science Building, 75 University Ave. W., Waterloo, ON, Canada; 8 Dalla Lana School of Public Health, University of Toronto, 6th Floor, Health Sciences Building, 155 College Street, Toronto, ON, Canada; 9 Centre for Research on Inner City Health, St. Michael's Hospital, 30 Bond St, Toronto, ON, Canada; 10 Department of Psychiatry, University of Toronto, 250 College Street, Toronto, ON, Canada

## Abstract

**Background:**

Community-based organizations (CBOs) are important stakeholders in health systems and are increasingly called upon to use research evidence to inform their advocacy, program planning, and service delivery efforts. CBOs increasingly turn to community-based research (CBR) given its participatory focus and emphasis on linking research to action. In order to further facilitate the use of research evidence by CBOs, we have developed a strategy for community-based knowledge transfer and exchange (KTE) that helps CBOs more effectively link research evidence to action. We developed the strategy by: outlining the primary characteristics of CBOs and why they are important stakeholders in health systems; describing the concepts and methods for CBR and for KTE; comparing the efforts of CBR to link research evidence to action to those discussed in the KTE literature; and using the comparison to develop a framework for community-based KTE that builds on both the strengths of CBR and existing KTE frameworks.

**Discussion:**

We find that CBR is particularly effective at fostering a climate for using research evidence and producing research evidence relevant to CBOs through community participation. However, CBOs are not always as engaged in activities to link research evidence to action on a larger scale or to evaluate these efforts. Therefore, our strategy for community-based KTE focuses on: an expanded model of 'linkage and exchange' (*i.e*., producers and users of researchers engaging in a process of asking and answering questions together); a greater emphasis on both producing and disseminating systematic reviews that address topics of interest to CBOs; developing a large-scale evidence service consisting of both 'push' efforts and efforts to facilitate 'pull' that highlight actionable messages from community relevant systematic reviews in a user-friendly way; and rigorous evaluations of efforts for linking research evidence to action.

**Summary:**

Through this type of strategy, use of research evidence for CBO advocacy, program planning, and service delivery efforts can be better facilitated and continually refined through ongoing evaluations of its impact.

## Background

Strategies for promoting evidence-based medicine have been well established in the literature [1-6], and efforts for facilitating the use of research evidence among health system managers and policymakers have been increasingly articulated in recent years [7-13]. Unfortunately, there have been few visible efforts, such as those developed for health system professionals, managers, and policymakers, to support the use of research evidence in community-based organizations (CBOs). By CBOs we mean not-for-profit organizations such as non-governmental, civil society organizations, or other grassroots organizations, overseen by an elected board of directors and guided by a strategic plan developed in consultation with community stakeholders. This is disappointing because CBOs constitute important health system stakeholders as they provide numerous, often highly valued programs and services to the members of their community, who are often marginalized and/or stigmatized members of society (*e.g*., people living with HIV/AIDS, and/or with mental health and addictions issues). Therefore, in order for CBOs to more effectively link research evidence to action in health systems and to strengthen the health systems in which they work, there is a need to better support their efforts to find and use research evidence. While we recognize that research evidence is only one input into the varied and complex decision-making processes of CBOs, it can play an important role in strengthening the effectiveness of their work.

In order to support the use of research evidence by CBOs, our primary objective is to develop a strategy for community-based knowledge transfer and exchange (KTE) that helps CBOs more effectively link research evidence to action. To address this goal, our specific objectives are: to outline the primary characteristics of CBOs, and why they are important stakeholders in health systems; to outline the concepts and methods of community-based research (CBR) and KTE; to compare the potential of CBR to link research evidence to action to those efforts more commonly discussed in the KTE literature; and to develop a strategy for community-based KTE that builds on both the strengths of CBR and existing KTE frameworks.

## Discussion

### What are CBOs?

The terminology used to describe CBOs can be quite diverse. The terms civil society organizations, grassroots organizations, and non-governmental organizations are commonly used to refer to the same or similar type of organization. In addition, these descriptors may vary based on the sector or 'community' that CBOs serve (*e.g*., 'AIDS service organizations' are often used in the HIV/AIDS sector in Canada). Furthermore, the notion of community and the organization of communities may be operationalized differently depending on the circumstances in which it is used [14]. For instance, Jewkes and Murcott (1998) analyzed how 'community' is operationalized in the context of identifying 'community representatives' for the purposes of achieving community participation. They found that 'community representatives' were often drawn from one small part of the voluntary sector [14]. In a context where community is limited to what Jewkes and Murcott (1998) call a voluntary sector 'elite', the notion of inclusive and democratized health systems decision-making may be compromised [14].

Despite the variability in the language used to describe community and CBOs, there are several descriptions in the literature relating to the core characteristics of 'community'. The most common and far reaching conceptions of 'community' relate to physical location or geographical areas (*e.g*., neighbourhoods) [15-19], common interests (*e.g*., values, norms, goals, or more specific attributes of a group such as gender or sexuality) [15-19], and joint action, activities, and patterned social interaction (*e.g*., volunteer activities and social networks) [16-19]. In addition, communities have also been described using a fourth characteristic that involves people organizing and interacting politically for the purpose of producing change [20]. Using many of these core characteristics, MacQueen *et al*. (2001) define community as 'a group of people with diverse characteristics who are linked by social ties, share common perspectives, and engage in joint action in geographical locations or settings.'

Using the above characteristics and definition of community as a guide, several basic characteristics of CBOs become evident. First, the roles of CBOs are often guided by a specific mission (*i.e*., an overall goal) that is shaped by commonly held values within the community that the CBO serves. Second, CBOs often have a governance structure consisting of board members that are elected from the members in the community. Third, they are typically not-for-profit organizations that are financed/funded through a combination of government and/or philanthropy (often from the communities they serve). Fourth, CBOs often deliver a specific set of programs or services that are shaped by the mission and values of the organization. Furthermore, many CBOs now have a growing interest in both using and conducting research (often in the form of CBR), with some CBOs explicitly incorporating a mandate to use and produce research evidence as part of their primary functions [21-23].

### Why are CBOs important stakeholders in health systems?

Calls for community involvement in health system activities can be found in a number of World Health Organization (WHO) strategies, including the Alma Ata Declaration, *Health for All by 2000*, *Health 21: Health for all in the 21*^st^*Century *[24], and the healthy cities initiative [25]. The Declaration of Alma Ata was unanimously adopted by all WHO member countries in 1978 with the WHO recently re-affirming its commitment to it in 2008 [26]. The Declaration states that:

'primary health care is essential health care based on practical, scientifically sound and socially acceptable methods and technology made universally accessible to individuals and families in the community through their full participation and at a cost the community and country can afford to maintain at every stage of their development in the spirit of self-reliance and self-determination' [27].

Further, the Declaration states that the people have a right and duty to participate individually and collectively in the planning and implementation of their healthcare [27]. Similarly, the strategies and agreements that have been based on the Alma-Ata Declaration -- *Health for All by 2000*, the *Ottawa Charter for Health Promotion *and *Health for All in the 21*^st^*Century *-- state in their key strategic principles that in order to 'close the gaps' in health (*i.e*., reduce health inequalities) community action needs to be strengthened, inter-sectoral collaboration among stakeholders is needed and communities and CBOs need be included as key policy stakeholders [24,28].

In addition to these international and national health strategies, WHO's healthy cities initiative also promotes inter-sectoral participation of communities and CBOs for achieving the *Health for All *strategies at the local level [25]. By including CBOs, it has been argued that delivery of basic health services (specifically in low-income countries) and accountability for public systems of providers can be improved [29]. In sum, CBOs are increasingly being asked to play important roles in health systems throughout the world, and there is a need to help them in this work by supporting their use of research evidence.

### CBR -- A brief overview of concepts and methods

Community-driven research initiatives are emerging as a useful source of research evidence for CBOs. Specifically, CBR (the terms action research, participatory research, and community-based participatory research are also commonly used in the literature) is rapidly emerging as an approach for addressing the complex health, social, and environmental problems that CBOs often address in their advocacy, program planning, and service delivery efforts [21,30-34].

Perhaps as a corollary to the growing interest in CBR from CBOs and academics in an increasing number of countries, there are a number of definitions available in the published literature [30,32,35-37]. One very popular definition, frequently cited in health-related literature, comes from Minkler and Wallerstein (2003) who define community-based participatory research as a:

'...collaborative approach to research that equitably involves all partners in the research process and recognizes the unique strengths that each brings. [Community-based participatory research] begins with a research topic of importance to the community with the aim of combining knowledge and action for social change to improve community health and eliminate health disparities' [30].

It is evident from this definition (and others in the literature) that three interrelated core principles or tenets characterize CBR as a unique approach to research: full participation in research processes by community members; producing relevant research evidence; and ensuring action is spurred by study findings [38]. In addition to these three principles, Minkler (2005) notes that 'individual, organizational, and community empowerment also is a hallmark of this approach to research' [38].

As can be seen, CBR is a 'user driven' and action-oriented approach to research (*i.e*., focused on influencing policy, and practice) that was originally developed to 'emphasize the participation, influence, and control by non-academic researchers in the process of creating knowledge and change' [32]. The primary argument in support of these efforts to foster collaborative and equitable partnerships with members of the community is that their inclusion helps increase the relevance of the research evidence produced, which has been demonstrated in a number of CBR studies involving marginalized populations [39-42]. With more relevant research evidence produced by incorporating local priorities from the outset, the effectiveness of health system planning and reform efforts can potentially be increased and time and money ultimately saved [34].

A good example of the importance of promoting collaboration and partnerships with community comes from the HIV/AIDS sector under the Greater Involvement of People Living with HIV/AIDS (GIPA) principle [43,44], which 'has evolved into a broad philosophy meant to underpin all forms of intervention (prevention, treatment, support, policy, and research) with persons living with HIV/AIDS' [22]. In the context of CBR, greater involvement of people living with HIV/AIDS can be operationalized in various ways, such as shared decision-making power with researchers or incorporating research skill building for people living with HIV/AIDS as a goal in CBR projects [22]. Implementing the GIPA principle through mechanisms such as these has been shown to result in enhanced credibility of community-based AIDS service organizations as policy actors [45], as well as reduced stigma and isolation [46] and increased feelings of personal empowerment and self-worth for people living with HIV/AIDS [47,48].

The CBR approach is also starting to gain recognition on a larger scale with major research funders such as the National Institutes of Health, the Agency for Healthcare Quality and Research, and the Centers for Disease Control in the United States, as well as the Canadian Institutes of Health Research and the Social Sciences and Humanities Research Council of Canada, now providing funds for general operating grants as well as capacity-building in support of community-academic partnership development [49-53]. In addition, Science Shops, which were originally developed in the Netherlands in the 1970s, have emerged as important community driven entities throughout the world (*e.g*., in central and eastern Europe and in China) that 'provide independent, participatory research support in response to concerns experienced by civil society'[54,55].

### KTE -- A brief overview of concepts and methods

There are many terms available for what we call KTE or more generally, putting knowledge into action [56,57]. For instance, Straus *et al*. (2009) indicate that the terms implementation science and utilization are often used in the UK and Europe, and dissemination or diffusion are commonly used in the US [57]. In Canada, the Canadian Institutes of Health Research, which is the country's largest funding body for health related research, uses the term knowledge translation and defines it as 'the exchange, synthesis, and ethically-sound application of knowledge -- within a complex system of interactions among researchers and users -- to accelerate the capture of the benefits of research for Canadians through improved health, more effective services and products, and a strengthened health care system'[58]. However, as Straus *et al*. note, despite the differing terminology, the core theme or goals that ties them together is moving beyond simple and passive dissemination of research evidence to more effectively facilitate its actual use [57].

While this is an important goal, efforts to link research evidence to action face many challenges. Specifically, Lavis *et al*. (2006) identify four primary challenges related to linking research evidence to action: research evidence competes with many other factors in decision-making processes; decision-makers may not value research evidence as an information input into decision-making processes; available research evidence may not be relevant for certain audiences; and research evidence is not always easy to use [59]. However, through a multi-faceted and interactive KTE strategy, the latter three challenges can be addressed in order to allow research evidence to play a stronger and more prominent role in decision-making processes (*i.e*., to help address the first challenge).

Lavis *et al*. (2006) provide a helpful framework for developing such a KTE strategy that addresses the challenges outlined above. The framework consists of four primary methods for linking research evidence to action: fostering a culture that supports the use of research evidence (*i.e*., within the target audience); producing research evidence that is relevant to the target audience; undertaking a range of activities for linking research evidence to action ('producer push,' facilitating 'user pull,' 'user pull' and 'exchange'); and evaluating efforts to link research evidence to action.

The first element of the framework -- fostering a culture for research evidence -- helps to ensure that target audiences are not only receptive to the idea of using research evidence in their decision-making but also place value on using it in their decision-making. If target audiences are receptive to using research evidence and place value on it as an input into decision-making, it is more likely that efforts to produce relevant research evidence and to disseminate it through integrated strategies (*e.g*., 'producer push' efforts or efforts to facilitate 'pull') will be successful in linking it to action.

In the second element of their framework, Lavis *et al*. (2006) highlight the notion that there needs to be research evidence available that is relevant to the topics and issues that decision-makers are addressing in their work (*e.g*., CBOs in the HIV/AIDS sector may require research evidence about how to organize an HIV prevention program in their community). The production of relevant research evidence can be supported through activities such as priority setting processes that involve target audiences and developing research funding calls based on the priorities identified. Examples of priority setting for research include the Listening for Direction consultation process for health services and policy issues that is conducted with national healthcare organizations in Canada every three years [60], or involving patients or patient representatives in the planning or development of healthcare [61-64] and in setting health system research agendas [65-67]

In addition to producing relevant research evidence, there is a need to ensure that it is likely to yield reliable actionable messages wherever possible [7]. A viable option for achieving this is conducting systematic reviews because they analyze the global pool of knowledge in a particular topic area. As a result, reviews constitute a more efficient use of time for research users because all information on a specific topic has already been identified, selected, appraised, and synthesized in one document [59]. Systematic reviews also offer a lower likelihood of providing misleading findings than other forms of research (*e.g*., a single observational study) and provide increased confidence in the findings due to the gains in precision that are obtained through synthesis of multiple studies [59]. In addition to these benefits, methods for systematic reviews are rapidly expanding (*e.g*., realist synthesis, meta-ethnography, or, more generally, syntheses of qualitative evidence), which allows for the incorporation of a broader spectrum of research evidence [68-75]. While the methods for syntheses of qualitative evidence are still developing, their production has increased in recent years with the Cochrane Qualitative Research Methods Group's reference database of qualitative reviews now providing references to over 360 syntheses [76]. Consequently, reviews are now better able to answer a broader spectrum of questions that may be asked in health systems (*i.e*., beyond questions of effectiveness) such as cost-effectiveness, and relationships and meanings, which increases their relevance to a broader range of target audiences (*e.g*., CBOs and health system managers and policymakers) [59,77].

The third element of the framework focuses on activities for linking research evidence to action, which includes four primary strategies that can be employed to produce a multi-faceted approach: 'producer push' efforts (*i.e*., producers of research disseminating findings to target audiences); efforts to facilitate 'user pull' (*i.e*., making research evidence available for target audiences when they identify the need for it); 'user pull' mechanisms (*i.e*., target audiences incorporating prompts for research evidence in their decision-making processes and developing their capacity to find and use research evidence); and 'exchange' efforts whereby the producers and users of researchers engage in a process of asking and answering questions together (*i.e*., building partnerships and working collaboratively in all stages of the research process, from the setting of research priorities, to conducting research, and linking findings to action). As can be seen, the fourth strategy of 'exchange' is also relevant to fostering a culture for research (*e.g*., engaging research users in the origination of an idea, proposal development, research conduct, and dissemination may increase the value they place on research) and in the production of relevant research evidence (*e.g*., through priority setting activities) [11,78,79].

Further building on 'push' efforts for linking research evidence to action, there are several steps to work through in order to effectively employ these efforts, which include identifying: the types of messages to be transferred and where they should be drawn from (*i.e*., systematic reviews, single studies or a combination); the target audience (to ensure the messages from research are presented in a way that is meaningful to them); credible messengers (a trusted messenger may have greater access to or influence among target audiences); and optimal processes and communications structures for delivery of key messages (*e.g*., providing a database that is searchable based on terms that are meaningful and relevant to the target) [7].

The last aspect of the framework is evaluating our efforts to link research evidence to action in order to determine which aspects of the strategy work (or don't), how and under what conditions. Without rigorous evaluations of efforts to link research evidence to action, we are left with anecdotal or indirect evidence about what works in KTE, which limits future efforts to modify, refine, and increase the effectiveness of our strategies.

### Similarities between CBR and KTE

While KTE is largely about harnessing existing research evidence and CBR is mostly concerned with generating new evidence, the approaches have many similarities with respect to their methods for linking research evidence to action, especially the importance placed on partnerships before, during, and after research initiatives. In order to further illuminate these similarities, we compare the four methods from the KTE literature (with examples) for linking research evidence to action, to examples of common approaches used by CBR. In doing so, we draw on examples from Canada's HIV sector and, to a lesser extent, from other jurisdictions.

As can be seen from Table 1, CBR and those involved in it (*i.e*., CBOs, researchers, research funders) may employ a number of strategies for linking research evidence to action within the four methods outlined from the KTE literature. Given that CBR encourages partnerships between researchers and community, it is not surprising that this helps to foster a culture that supports the use of research evidence, especially if it is relevant to the needs and priorities of a community. In contrast, we can see that CBR, with the exception of 'exchange' efforts, lacks coordinated large scale efforts that attempt to provide actionable messages from a large pool of knowledge or that attempt to reach beyond the specific community (or individual study) on which a study was focused.

**Table 1:** Comparison of knowledge transfer and exchange (KTE) activities and community-based research (CBR) methods/community-based organization (CBO) initiatives for linking research evidence to action

Types of KTE Activities	Examples of KTE Activities	Examples of CBR methods and CBO initiatives
**Fostering a culture that supports research use**	▪ Some funders require ongoing 'linkage and exchange' (*i.e*., producers and users of research evidence work collaboratively on proposal development and research conduct) (*e.g*., the Canadian Health Services Research Foundation).	▪ CBR projects may use community advisory committees to engage community members in guiding the research process and the dissemination of the results.
	▪ Trusted researchers or knowledge brokers periodically highlight the value of research evidence (*e.g*., highlighting positive examples of research use in practice or decision-making).	▪ Some conferences that address issues of community interest develop strategies to include community members (*e.g*., Community-Campus Partnerships for Health (CCPH) in the U.S.).
	▪ Some funders provide grants for linking research evidence to action.	▪ Community members often play the role of co-principal investigator in CBR, which helps to foster a sense of leadership, responsibility, and ownership of the research.

**Production of research to key target audiences**	▪ Some funders engage in priority setting with key target audiences to ensure that systematic reviews and primary research address relevant questions (*e.g*., the Listening for Direction priority setting process for health services and policy research from the Canadian Health Services Research Foundation) [60].	▪ Some CBR funders and intermediary organizations periodically organize multi-stakeholder 'think tanks' to develop a research agenda through consensus.
	▪ Some funders commission scoping reviews or rapid assessments of the literature to identify important gaps for targeted research funding.	▪ CBOs, researchers, research funders, and government periodically form task forces related to specific areas of interest for the purpose of coordinating action on community generated research agendas.
	▪ Some researchers involve members of the target audiences in the research process.	▪ CBR requires partnerships between researchers and community during all phases in the research process in order to ensure relevance and sensitivity to community concerns.
	▪ Some networks of systematic review producers commit to updating them regularly (*e.g*., the Cochrane Collaboration).	▪ Some CBR funders offer 'enabling' or 'seed' grants to assist in question identification, partnership development and protocol development.

**Activities to link research to action**		
**'Push'**	▪ Some organizations provide email updates that highlight actionable messages from relevant and high quality systematic reviews (*e.g*., SUPPORT summaries) [83].	▪ Some organizations or associations develop websites/databases and listservs dedicated to highlighting research originating in and undertaken through community-university partnerships (*e.g*., CCPH).
	▪ Researchers, funders or knowledge brokers will periodically engage in capacity building and consultations with research users to enhance their ability to undertake evidence-informed push efforts that meet the needs of their target audiences.	▪ Researchers, funders or knowledge brokers sometimes disseminate fact sheets or newsletters to highlight results from specific studies or about a specific topic of interest (*e.g*., The Ontario HIV Treatment Network in Canada and CCPH in the U.S.).
		▪ CBR partners often initiate community forums to present research results.
		▪ Academic (and increasingly community) partners involved with CBR often present at conferences and publish in journals.

**Facilitating **'**pull'**	▪ Some groups provide 'one stop shopping' websites that provide user-friendly and high quality systematic reviews relevant to specific target audiences (*e.g*., Health Systems Evidence)[84].	▪ Some CBR projects develop websites to profile their research evidence and provide resources that they have produced as part of their research (*e.g*. the Positive Spaces Healthy Spaces housing project in Canada) [85].
	▪ Researchers, funders or knowledge brokers sometimes undertake capacity building with key target audiences to help better acquire, assess, adapt, and apply research evidence (*e.g*., WHO sponsored workshops to help policymakers find and use research evidence).	▪ Some organizations or associations develop websites/databases and listservs dedicated to highlighting research originating in and undertaken through community-university partnerships (*e.g*., CCPH).
		▪ Some funders of CBR offer capacity-building resources to bring together community stakeholders for skill-building activities.

**'Pull'**	▪ Some research users will design prompts in the decision-making to support research use	▪ Some CBOs incorporate prompts to research evidence into their strategic goals or values (*i.e*., incorporating organizational structures/processes for using evidence).
	▪ Some research users will conduct self-assessments of their capacity to acquire, assess, adapt, and apply research and engage in capacity building activities in these areas.	

**'Exchange'**	▪ Researchers and research users build partnerships and work collaboratively in setting research priorities, conducting research and linking research to action.	▪ CBR methods and CBR funders require partnerships between researchers and community during all phases in research in order to ensure its relevance (*i.e*., topics and outcomes measured) and sensitivity to community concerns and to facilitate eventual use of the results (*e.g*., specific funding calls from the National Institutes of Health in the U.S., the Canadian Institutes of Health Research and the Social Sciences and Humanities Research Council in Canada).

**Evaluation**	▪ Some researchers and research funders evaluate the effectiveness of their efforts (*i.e*., one or more of the activities outlined above) for linking research evidence to action.	▪ CBR projects sometimes engage target audiences in reflection processes about the specific impacts the project had (*e.g*., was quality of life enhanced? If so, how?)

Acronyms used: CBO = community-based organizations, CBR = community-based research, KTE = knowledge transfer and exchange, CCPH = Community-Campus Partnerships for Health,

### Strengths and limitations of CBR for linking research evidence to action

Based on this comparison, it appears as though CBR is more effective in some of the areas for linking research evidence to action than others. In Table 2, we present, based on the common approaches outlined in Table 1, areas where CBR is particularly strong at linking research evidence to action and areas where it appears to be limited in its reach, in order to help identify domains for strategic expansion.

**Table 2:** Strengths and limitations of community-based research (CBR) for linking research to action

Types of KTE Activities	CBR strengths	CBR limitations
**Fostering a culture that supports research use**	▪ Funding typically requires partnerships between researchers and community members and/or CBOs (*e.g*., funding calls from the National Institutes of Health in the U.S., the Canadian Institutes of Health Research and the Social Sciences and Humanities Research Council in Canada).	▪ Scope of partnerships often limited as community partners are often those that already have a culture that supports the use of research evidence.
	▪ Emphasis on capacity building and actionable outcomes resonates well with the grass roots orientation of many CBOs.	▪ Often no dedicated funding for linking CBR to action (as opposed to funding to conduct the research).
		▪ The process-oriented nature of CBR can push a project beyond initial timelines, limiting the ability of some partners to remain engaged long-term.
		▪ Those who have the most influence on CBO culture (*e.g*., Executive Directors) are not always included as the community partner from a CBO.

**Production of research to key target audiences**	▪ CBR projects are often developed through consultation with local communities in order to ensure they are addressing community relevant issues and needs.	▪ CBR projects typically take the form of single locally-based studies and not systematic reviews of studies conducted across a range of communities.
		▪ CBR projects are not typically written up in a way that puts the findings in the context of the global pool of knowledge.

**Activities to link research to action**		
**'Push'**	▪ Dissemination of actionable messages is often strong at the local level through the use existing networks and partnerships.	▪ Actionable messages derived from CBR projects often not shared on a larger scale (*i.e*., outside the communities in which the CBR projects were conducted) despite their potential broader applicability.
		▪ 'Push' efforts in communities limited to projects conducted locally (*i.e*., potentially informative projects from other communities are not actively 'pushed' to relevant target audiences).
		▪ Minimal capacity building designed specifically for enhancing 'push' efforts.

**Facilitating **'**pull'**	▪ Capacity-building for research within communities and CBOs through participation in CBR projects is a central goal of the CBR approach.	▪ No capacity building in acquiring, assessing, adapting, and applying research evidence.
		▪ Few 'one-stop shopping' websites or resources exist that provide user-friendly, high-quality, and community--relevant research evidence (*e.g*., CBR and/or community-relevant systematic reviews) with the actionable messages clearly identified.

**'Pull'**	▪ Some CBOs and communities are effective at identifying research needs and partnering in CBR projects or seeking out research evidence.	▪ CBOs typically don't have in place mechanisms to prompt them to review their programming in light of the available research evidence (either on a rotating basis for select programs or all at once during strategic planning).
		▪ Smaller CBOs do not always have the capacity, resources or time to acquire, assess, adapt and apply research evidence in their settings.

**'Exchange'**	▪ Equitable partnerships between community, researchers and other stakeholders are a core requirement of the CBR approach.	▪ Scope of partnerships often limited to the same researchers and community partners in many projects. Many not representative of the breadth of perspectives in the community.
		▪ Other stakeholders (*e.g*., healthcare managers and policymakers not always sought (or available) for partnerships.

**Evaluation**	▪ Some projects have systematically evaluated the types of topics previously addressed by CBR and the quality of those projects in order to inform future research and funding initiatives [31].	▪ Minimal efforts in the community sector to evaluate the impact of CBR and other community-based KTE strategies on action beyond those communities most directly involved in the CBR.
		▪ If evaluations of the impact of research are completed, they may be done by the researchers of the study, thereby introducing a source of bias.

Acronyms used: CBO = community-based organizations, CBR = community-based research, KTE = knowledge transfer and exchange

As can be seen in Table 2, CBR has a number of strengths for linking research evidence to action at the local level, especially for fostering a culture that supports the use of research evidence, production of relevant research evidence, and 'exchange' activities. We can see that the emphasis placed upon partnerships between researchers and community helps to foster a culture that supports the use of research evidence within those CBOs involved in CBR. It also supports the production of relevant research evidence by ensuring that CBR projects address issues that are important to the community while remaining sensitive to their needs. Furthermore, the community networks and partnerships developed through CBR help with 'push' efforts targeting the local level. CBO and community participation in CBR also provides important opportunities for capacity building, which helps to facilitate user 'pull' because they are better equipped to acquire, assess, adapt, and apply research evidence in their settings.

Although CBR does exhibit several strengths, there are also several limitations that are apparent. For example, in Table 2 we point out that the scope of partnerships with CBOs and community may be limited to those that already have a culture that supports the use of research evidence. As such, the research priorities developed through these partnerships may not accurately reflect the needs of the target audience. An additional limitation that emerges from Table 2 is the mix of research evidence being produced and its impact on the actionable messages that can be derived. CBR is often focused on the production of single, locally-based studies and does not typically synthesize global pools of knowledge on community issues in order to provide actionable messages to a broader audience. This does not mean that single CBR studies are unimportant, because they offer high utility by providing locally applicable information to CBOs, community, and researchers. Our contention is that these studies could be complemented by syntheses of research evidence on community relevant issues because they would help determine whether questions have already been answered in similar communities, allow participants to learn about the strengths and weaknesses of approaches that have previously been used, and would put results in the context of the global pool of knowledge (resulting in actionable messages that have broader applicability). Therefore, while CBR does offer very promising prospects for linking research evidence to action, there is a need to consider expanding these efforts to a larger scale, complementing single CBR studies with syntheses and by expanding KTE activities (*i.e*., 'push', efforts to facilitate 'pull,' and 'pull').

### A framework for community-based KTE

In Table 3, we provide an outline for additional activities that are intended to build upon and complement current CBR efforts for linking research evidence to action. Our proposed framework focuses on four primary areas: developing and maintaining partnerships; increasing the production of community relevant systematic reviews; creating an integrated and large-scale evidence service; and evaluating efforts to undertake CBR and to link research evidence to action. First, across the spectrum of the framework, we maintain CBR principles by placing emphasis on partnerships between researchers, CBOs, community members, and other stakeholders through a model of 'linkage and exchange.' Maintaining these principles is important because it not only helps to ensure the production of 'user driven' relevant and action-oriented research evidence but also helps to position CBOs as policy actors in health system decision-making forums where they may not normally be included.

**Table 3:** Framework for additional activities for community-based research (CBR) to link research to action

Types of KTE Activities	Proposed Additional Activities for CBR
**Fostering a culture that supports research use**	▪ Through an ongoing model of 'linkage and exchange', engage CBOs in the development, production and updating of community relevant systematic reviews in order to help increase their perceived value as an input to CBO decision-making.
	▪ Widen the scope of CBR partnerships by seeking out new key stakeholders in the community (*e.g*., knowledge brokers facilitating partnerships with stakeholders that are interested in addressing similar issues).
	▪ Provide dedicated funds for projects that attempt to link CBR to action on a large-scale (*i.e*., not only within local communities but also across jurisdictions at the provincial/state, national and international level).
	▪ Within an evidence service that identifies actionable messages from research evidence (see activities for 'push' and facilitating 'pull' below), periodically highlight case studies where research was successfully used in a community setting to inform CBO advocacy, program planning or service provision.

**Production of research to key target audiences**	▪ Researchers and funders engage CBOs in priority setting processes for CBR studies in areas where there is minimal research, for systematic reviews in areas where there is pool of research evidence already accumulated, and for developing systems to link research evidence to action at the community level.
	▪ Produce targeted funding streams based on priority setting with CBOs for CBR, community-relevant systematic reviews and initiatives to develop systems to link research evidence to action at the community level.
	▪ Engage CBOs in the development, production and updating of systematic reviews in order to ensure they produce evidence that is relevant.

**Activities to link research to action**	
**'Push'**	▪ Develop an evidence service that identifies actionable messages for communities from relevant systematic reviews and involve credible messengers in providing them to CBOs in user-friendly formats (*e.g*., short, structured summaries with graded entry to the full details of the review).
	▪ Engage CBOs to develop a 'push' evidence service with a stream of community relevant systematic reviews (or CBR projects where reviews are not available).

**'Pull'**	▪ Conduct periodic capacity-building initiatives with CBOs to help them identify areas where research can be used as an input into their decision-making.
	▪ Periodically highlight instances where the use of research evidence made the difference between success and failure of a CBO initiative.

**Facilitating **'**pull'**	▪ Create an evidence service, in combination with 'push' efforts, that provides 'one stop shopping' websites/databases of relevant and user-friendly systematic reviews with actionable messages that can be located through search terms that are relevant to CBOs.
	▪ Provide capacity-building to CBOs to help build their skills related to acquiring, assess, adapting and applying research evidence in their organization.

**'Exchange'**	▪ Engage CBOs in deliberative dialogues where health system stakeholders gather to discuss a pre-circulated evidence brief and have 'off-the-record' deliberations (*e.g*., the McMaster Health Forum).
	▪ Engage CBOs in the development, production, and updating of systematic reviews in order to build and maintain partnerships between relevant stakeholders.
	▪ Use knowledge brokers and/or other credible messengers to promote additional partnerships with CBOs previously not engaged in CBR and other interested stakeholders.

**Evaluation**	▪ Researchers, CBOs, and funders work collaboratively to rigorously evaluate the impact of strategies to link research evidence to action such as those outlined above (*e.g*., evaluating the effectiveness of an evidence service for relevant and user-friendly systematic reviews that combines 'push' and efforts to facilitate 'pull').

Acronyms used: CBO = community-based organizations, CBR = community-based research, KTE = knowledge transfer and exchange

Second, we outline throughout the framework a greater emphasis on both producing and disseminating systematic reviews that address topics of interest to CBOs because they are more likely to provide reliable actionable messages than single research studies. Furthermore, systematic reviews can represent a more efficient use of time for busy CBOs because they provide a reliable assessment of an entire pool of knowledge on a given topic. Therefore, in Table 3, we outline various activities related to systematic reviews for fostering a culture of research (*e.g*., engaging CBOs in the conception, production and updating of reviews), generating community relevant reviews (*e.g*., priority setting processes for areas where reviews can be completed), activities to link research evidence to action (*e.g*., 'one stop shopping' websites/databases for community relevant systematic reviews and capacity building workshops designed to help CBOs find and use research evidence), and evaluation of efforts to link research evidence to action (*e.g*., evaluating the impact of 'one stop shopping' websites on the use of research evidence in CBOs).

The third area of focus for our framework is on developing a large-scale evidence service consisting of both 'push' (*e.g*., email updates to new and relevant systematic reviews) and efforts to facilitate 'pull' (*e.g*., a 'one stop shopping' database) that highlight the take-home messages (actionable messages where possible) from community relevant systematic reviews in a user-friendly way for CBOs (*e.g*., short, structured summaries that outline take-home messages, benefits, harms, and costs of the interventions, programs, or services addressed in a review). This type of evidence service will help ensure that CBOs have timely access to relevant and user-friendly systematic reviews either when they face decisions that could be informed by research evidence or when they are asked to participate in forums for health system strategizing and decision-making.

Finally, we propose that there is a need to develop collaborative and rigorous evaluation strategies that assess the impact of activities for linking research evidence to action to allow for ongoing refinement, modification, and expansion of KTE activities. This requires the implementation of a community-based KTE strategy, identification of relevant outcomes to be measured, availability of instruments to measure the desired outcomes, and rigorous study designs (*e.g*., randomized controlled trials with an accompanying qualitative process evaluation) for the evaluation process.

### Implications

Implementing a strategy such as this would build on important KTE structures and processes that have been previously implemented or are in the process of being implemented internationally for other stakeholders. For example, promising KTE services that integrate a number of the activities for linking research evidence to action that we present here are in development through two regional initiatives in low- and middle-income countries -- the Regional East African Community Health (REACH) Policy Initiative and the WHO-sponsored Evidence Informed Policy Networks emerging in the Western Pacific, Africa, the Americas, and the Eastern Mediterranean [59,80]. Similarly, from the clinical sector, Evidence Updates [81] and McMaster PLUS [5] are good examples of evidence services that disseminate high-quality and high-relevance studies at both the global and regional levels. In addition, results from a cluster randomized controlled trial of McMaster PLUS lends support to the idea of creating an integrated evidence service (*i.e*., one that combines 'push,' efforts to facilitate 'pull' and 'exchange') because increases in clinicians' utilization of evidence-based information from a digital library have been found [6].

By building upon existing KTE frameworks and developing this strategy for community-based KTE, we have taken an important step towards recognizing the important roles that CBOs' advocacy, program planning, and service delivery can play in health systems at the international, national, and local levels. In addition, it provides a practical outline for how to expand upon the existing efforts of those engaged in CBR in order to better support the research needs of CBOs. Such a strategy will help CBOs draw upon research evidence when engaging in international, national, and local healthcare system strategies, delivery, and decision-making.

Despite this, there are some potential criticisms and limitations that could be levied against the development of our framework and the framework itself. First, the derivation of our framework by comparing CBR to KTE and then drawing lessons from KTE is often based heavily on the Canadian context (although not exclusively). However, based on the fact that CBR is recognized by many funders and organizations outside of Canada (*e.g*., the National Institutes of Health, Centres for Disease Control and Prevention, Agency for Healthcare Research and Quality, and 'science shops' that are located in numerous countries around the world), we feel that our descriptions and conclusions are relevant to other communities that are similarly engaged in CBR.

With respect to the framework itself, there are two potential limitations that are apparent. First, eventual implementation of our framework rests on the idea that there are (or will be) community-relevant systematic reviews available to build an evidence service. We believe that this limitation can be addressed through effective priority setting processes with CBOs, such as those in place for health system managers and policymakers [8,79], and through targeted funding streams and/or commissioning of research that address these priorities. Second, our proposal to place increased emphasis on systematic reviews could be argued to diminish the value of CBR and its grass roots approach. While recognizing this concern, we are not proposing that systematic reviews are the only source of research evidence. For instance, the actionable messages that may be derived from systematic reviews could be used in conjunction with locally applicable CBR studies and/or local data. In addition, CBR studies will continue to provide relevant and locally applicable research evidence where no reviews exist.

### Future Research

Our framework provides multiple opportunities for future research initiatives. First, in order to allow for timely evaluation, there is a need to develop methods for evaluating the impact of the activities outlined in our framework. Second, there is a need for ongoing priority setting processes for systematic reviews that address the research needs of CBOs. Third, those involved in systematic review production can begin to partner with CBOs and produce reviews based on the priorities identified in order to continually build a stream of reviews to use in a future community targeted evidence service. Fourth, there is a need for in-depth consultation with CBOs in various sectors to determine the types of information that should be highlighted in user-friendly summaries of systematic reviews and optimal formats for the summaries (*e.g*., 1:3:25 format -- one page of take-home messages, three-page executive summary, and 25 page report) [82]. Lastly, in-depth consultation about how to categorize and assess the relevance of reviews is needed before our framework can be operationalized.

## Summary

With a growing need to make relevant and user-friendly research evidence available to CBOs in order to support their advocacy, program planning, and service delivery functions in international, national, and local health systems, we have developed a strategy for community-based KTE that will help CBOs more effectively link research to action at the community level.

CBR provides a useful source of research evidence as well as tools for linking research to action for CBOs, and the KTE literature provides helpful existing frameworks that can be used to determine strategic areas to help expand upon CBR to develop a strategy for community-based KTE.

CBR provides several useful tools and strategies for linking research evidence to action (*e.g*., fostering a culture that supports the use of research evidence, promoting the production of relevant research evidence, and disseminating it through processes of 'exchange'), but it is limited in the scale of its scope and activities and the activities employed for linking research evidence to action ('push,' efforts to facilitate 'pull,' 'pull,'and 'exchange') are similarly limited in the scope of the target audience reached and the type of research and actionable messages transferred (*i.e*., focused on single studies, as opposed to syntheses that may have greater applicability across communities).

Our strategy for community-based KTE focuses on: an expanded model of 'linkage and exchange'; a greater emphasis on both producing and disseminating systematic reviews that address topics of interest to CBOs; developing a large-scale evidence service consisting of both 'push' efforts and efforts to facilitate 'pull' that highlights actionable messages from community relevant systematic reviews in a user-friendly way; and rigorous evaluations of efforts for linking research evidence to action.

Future research and initiatives in this area should focus on: developing methods for evaluating the impact of the activities outlined in our framework; ongoing priority setting processes for systematic reviews that address the research needs of CBOs; continually build a stream of research evidence to use in a future community-targeted evidence service by having those involved in systematic review production partner with CBOs to produce reviews based on their priorities; and conduct in-depth consultation with CBOs in various sectors for determining the types of information that should be highlighted in user-friendly summaries of systematic reviews, optimal formats for the summaries, and how to categorize and assess the relevance of reviews.

## Competing interests

The authors declare that they have no competing interests.

## Authors' contributions

MGW contributed to the conception, design, wrote the original draft manuscript, and incorporated revisions from each of the co-authors. JNL contributed to the conception and design of the manuscript and provided revisions. RT contributed to the conception and design of the manuscript and provided revisions. SBR contributed to the conception and design of the manuscript and provided revisions. All authors read and approved the final manuscript.
